# Automated tube voltage selection in pediatric non-contrast chest CT

**DOI:** 10.1371/journal.pone.0204794

**Published:** 2018-10-03

**Authors:** Azadeh Hojreh, Peter Homolka, Jutta Gamper, Sylvia Unterhumer, Daniela Kienzl-Palma, Csilla Balassy, Thomas Wrba, Helmut Prosch

**Affiliations:** 1 Department of Biomedical Imaging and Image-guided Therapy, Medical University of Vienna, Vienna, Austria; 2 Centre for Medical Physics and Biomedical Engineering, Medical University of Vienna, Vienna, Austria; 3 Centre for Medical Statistics, Informatics and Intelligent Systems, Medical University of Vienna, Vienna, Austria; 4 IT-Systems & Communications, IT4Science, Medical University of Vienna, Vienna, Austria; Northwestern University Feinberg School of Medicine, UNITED STATES

## Abstract

**Background:**

Modern CT scanners provide automatic dose adjustment systems, which are promising options for reducing radiation dose in pediatric CT scans. Their impact on patient dose, however, has not been investigated sufficiently thus far.

**Objective:**

To evaluate automated tube voltage selection (ATVS) in combination with automated tube current modulation (ATCM) in non-contrast pediatric chest CT, with regard to the diagnostic image quality.

**Materials and methods:**

There were 160 non-contrast pediatric chest CT scans (8.7±5.4 years) analyzed retrospectively without and with ATVS. Correlations of volume CT Dose Index (CTDI_vol_) and effective diameter, with and without ATVS, were compared using Fisher’s z-transformation. Image quality was assessed by mean signal-difference-to-noise ratios (SDNR) in the aorta and in the left main bronchus using the independent samples t-test. Two pediatric radiologists and a general radiologist rated overall subjective Image quality. Readers’ agreement was assessed using weighted kappa coefficients. A p value <0.05 was considered significant.

**Results:**

CTDI_vol_ correlation with the effective diameter was r = 0.62 without and r = 0.80 with ATVS (CI: -0.04 to -0.60; p = 0.025). Mean SDNR was 10.88 without and 10.03 with ATVS (p = 0.0089). Readers’ agreement improved with ATVS (weighted kappa between pediatric radiologists from 0.1 (0.03–0.16) to 0.27 (0.09–0.45) with ATVS; between general and each pediatric radiologist from 0.1 (0.06–0.14) to 0.12 (0.05–0.20), and from 0.22 (0.11–0.34) to 0.36 (0.24–0.49)).

**Conclusion:**

ATVS, combined with ATCM, results in a radiation dose reduction for pediatric non-contrast chest CT without a loss of diagnostic image quality and prevents errors in manual tube voltage setting, and thus protecting larger children against an unnecessarily high radiation exposure.

## Introduction

Possible ionizing radiation effects in the young and growing bodies of children [1, 2] require that radiation exposure and scan protocols must be carefully adapted to size, age, and clinical needs [3–7]. To reduce radiation dose, modern CT scanners provide automatic dose adjustment systems, such as automated tube current modulation (ATCM) [8–11] and automated tube voltage selection (ATVS) [12–19].

CareDose4D (Siemens Healthineers, Siemens Healthcare GmbH, Germany) is an example of ATCM. CareDose4D assesses the cross-sectional size of the patient being scanned and adjusts tube current time product (mAs) relative to the reference tube current time product (reference mAs), which is set by the user for the selected examination and for an average-sized patient (70–80 kg for adults, 20kg for children), to provide adequate image noise [8]. The degree to which mAs is adjusted for patient size can be selected using “weak,” “average,” or “strong” compensation settings [8]. CareDose4D adjusts mAs over the patient’s z-axis and in the x- and y-axis based on the topogram, and modulates mAs in real-time, according to the attenuation measured at each tube angle by using feedback from the previous rotation during scanning (four dimensions) [8].

An example of ATVS is Care-kV (Siemens Healthineers, Siemens Healthcare GmbH, Germany). Care-kV, in combination with CareDose4D, uses attenuation information collected by the topogram and selects kV and modulates mAs maintaining the user-chosen image quality, based on the user-selected reference kV and reference mAs [12, 17, 20]. The user must select a contrast-strength setting, using a slider bar, based on the presence or absence of iodine in the study [19]. The slider provides 12 levels, so-called, dose saving optimized levels (DSO levels) depending on the diagnostic task [17].

While automatic tube current modulation [8–10] and automatic tube voltage selection in contrast-enhanced pediatric CT are well established, and their effectiveness has been proven [18, 19], there is no published research about the effect of automatic tube voltage selection in non-contrast pediatric CT.

Non-contrast chest CT is commonly used for patients suspected of having diffuse parenchymal lung diseases or bronchial diseases, such as cystic fibrosis (CF), and to monitor structural lung changes [21–24] before they become evident with other diagnostic tests [21]. Non-contrast CT is also applied to monitor patients after lung transplantation or in patients with contraindications to contrast agents, who require a chest CT, such as for (re-) staging in the case of malignancy.

The aim of this retrospective study was to evaluate the influence of ATVS, in combination with ATCM in non-contrast pediatric chest CT, on radiation dose, compared to a CT protocol in which tube voltage is set manually, based on patient weight, with regard to diagnostic image quality.

## Materials and methods

The institutional review board of Medical University of Vienna approved the study and waived the requirement for informed parental consent, because the study was a retrospectively data analysis. (IRB 855/2011). All procedures performed in the study involving human participants were in accordance with the ethical standards of the institutional review board and with the 1964 Helsinki declaration and its later amendments.

During an observation interval of three years, all pediatric non-contrast chest CT scans that were performed on a dual-source CT scanner (Somatom Definition Flash, Siemens Healthineers, Siemens Healthcare GmbH, Germany), were retrospectively analyzed with regard to radiation exposure and image quality, before and after ATVS implementation.

Examinations were divided into two groups:

- ATCM group: Examinations were carried out with an ATCM procedure (CareDose4D, Siemens Healthineers, Siemens Healthcare GmbH, Germany) only, before ATVS implementation.
- ATVS + ATCM group: Examinations were performed with an ATVS procedure (Care-kV, Siemens Healthineers, Siemens Healthcare GmbH, Germany) in addition to ATCM (CareDose4D, Siemens Healthineers, Siemens Healthcare GmbH, Germany), after ATVS implementation.

Scan parameters for both groups are listed in Table 1.

**Table 1:** Scan parameters of the ATCM group and of the ATVS +ATCM group.

Scan Parameters	ATCM group	ATVS + ATCM group
**Collimation**	2x64x0.6mm	2x64x0.6mm
**Flash spiral mode**	on	on
**Pitch**	3	3
**CareDose4D strength**	average	average
**Care-kV**	Not implemented	On (DSO 3)
**kV**	Fixed Setting	Reference kV: 100
- < 45 kg	80 kV	
- > 45 kg	100 kV	
**Reference mAs**	50mAs	80mAs @ 100kV

ATCM, automated tube current modulation; ATVS, automated tube voltage selection; DSO 3, dose saving optimized level 3 for non-contrast, as suggested by the manufacturer for non-contrast CT scans

For lung parenchyma evaluation, axial 1-mm slices with a 0.8-mm increment were reconstructed with a B60f kernel. The window settings were (width/center) 1500/-650 Hounsfield units (HU).

All patient image data were evaluated using an IEC (International Electrotechnical Commission)-, CE (Conformité Européenne)-, and FDA (Food and Drug Administration)-certified PACS (picture archiving and communication system, IMPAX DS 3000, Agfa Healthcare, Mortsel, Belgium) on a diagnostic gray-scale monitor (Barco MDCG-3120, Brussels, Belgium).

### Radiation exposure

Volume Computed Tomography Dose Index (CTDI_vol_) data were extracted from Digital Imaging and Communications in Medicine (DICOM)-structured reports from the PACS. Dose data from the children were pooled according to age groups (younger than 1 year, 1 to younger than 5 years, 5 years to younger than 10 years, and 10 years to younger than 15 years), in accordance with the recommended procedures of the International Atomic Energy Agency (IAEA) and the Austrian Standard Institute (ASI) for comparing children’s doses to dose reference levels using age-banding [25, 26]. To evaluate the effect of ATVS, CTDI_vol_ was considered a function of effective diameter. The effective diameter of each patient was calculated as $\sqrt{LAT.AP}$ [27], where LAT corresponds to the lateral and AP to the anterior-posterior diameter of each patient, measured at the widest area in the axial CT thoracic scans, which almost always corresponded to the thoracic diameter at the level of the 9th thoracic vertebral body.

Correlations of CTDI_vol_ with effective diameter in both groups were calculated. Confidence intervals for the differences in the correlations were calculated using Fisher’s z-transformation to determine whether dose values correlated better with the children’s size, with or without ATVS.

### Objective and subjective image quality analysis

Objective image quality measures were assessed by mean signal-difference-to-noise ratios (SDNRs), measured in the aorta (HU_aorta_ and standard deviation STD_aorta_) and in the left main bronchus (HU_air_). Signal-difference-to-noise ratios were defined as (HU_aorta_–HU_air_)/ STD_aorta_ and SDNRs were averaged from three consecutive thin-section CT slices reconstructed with a lung reconstruction kernel.

Two pediatric radiologists with seven and eight years experience, respectively, and a general radiologist with seven years experience, rated overall subjective image quality, blinded, using a 10-point scale. For grading the subjective image quality, four criteria were defined: motion artifacts; visibility of the subsegmental bronchial system; visibility of the lung interstitium; and visibility of the interlobar fissures. A 10-step scale (Likert scale) defined 9–10 as excellent quality, 7–8 as good quality, 5–6 as acceptable quality, 3–4 as poor quality, and 1–2 as non-diagnostic images.

### Statistical method

The objective image quality of both groups was compared using an independent samples t-test. The agreement between the three radiologists was assessed by weighted kappa coefficients with quadratic weights in order to account for rating disagreements accordingly. Based on Landis and Koch, the levels of readers’ agreement for the weighted kappa score were defined as 0–0.2 for slight agreement, 0.21–0.4 for fair agreement, 0.41–0.6 for moderate agreement, 0.61–0.8 for substantial agreement, and 0.81–1 for (almost) perfect agreement [28]. The coefficients and their 95% confidence intervals were calculated separately for ratings in both groups. Results with a p value <0.05 were considered significant. The statistical analysis was achieved using the software package R version 3.1.2 (The R Foundation for Statistical Computing, Vienna, Austria).

## Results

There were 160 children (91 male and 69 female; 8.7 ± 5.4 years) included in the study. Ninety-two children (51 male and 41 female; 8.7 ± 5.3 years) were examined with ATCM only, and 68 children (40 male and 28 female; 8.8 ± 5.6 years) with ATVS in combination with ATCM. The youngest child was two months and the oldest was 17 years. Patient weight was 29.1 ± 14.3 kg and patient height was 129.7 ± 29.2 cm in the ATCM group, and 28.7 ± 14.9 kg and 128.7 ± 30 cm in the ATVS + ATCM group. There was no significant difference in gender, age, weight, or height between the two study groups (p > 0.05).

**Fig 1:**
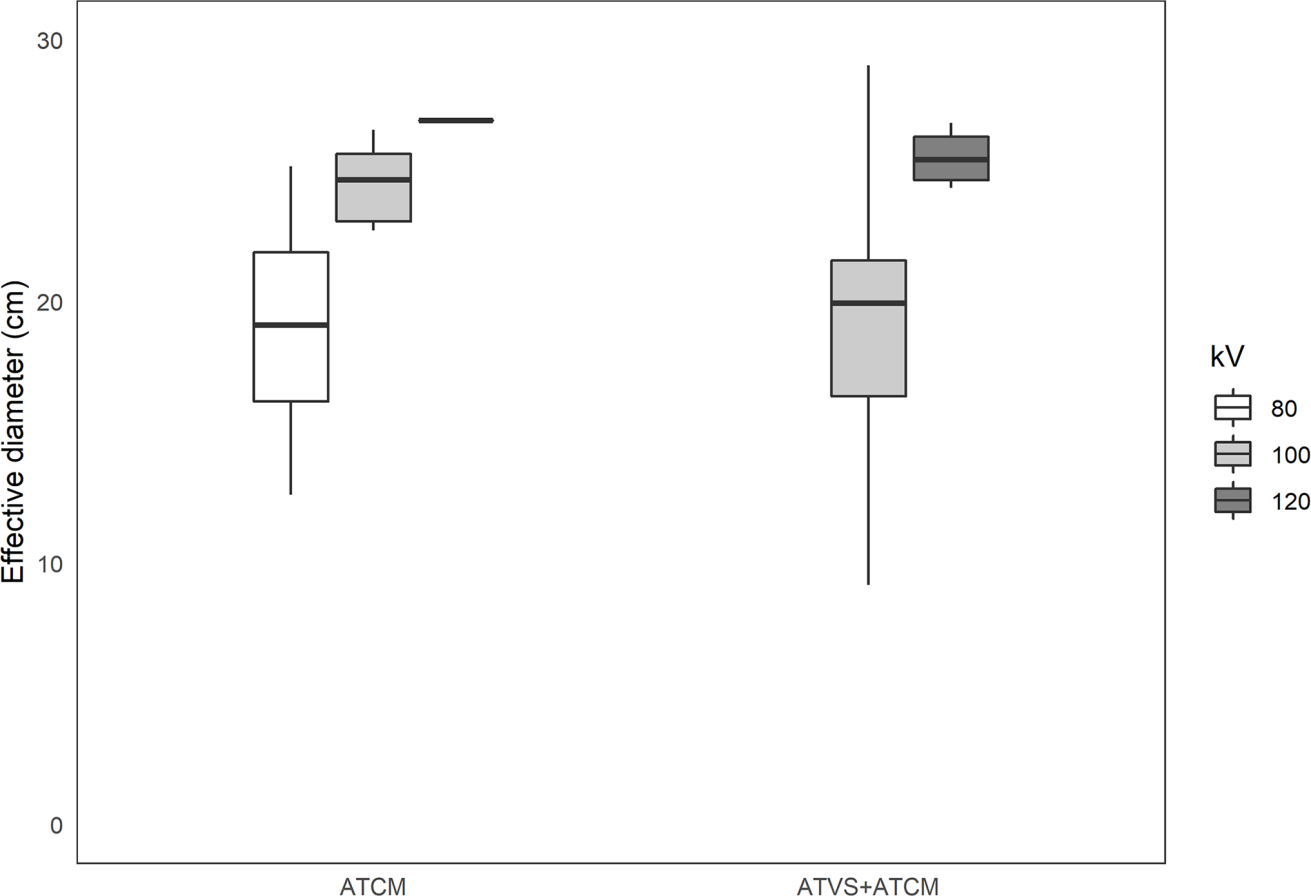
Boxplots of selected kV at effective diameter, in the ATCM group and in the ATVS + ATCM group.

In the ATCM group, following the department CT scan protocol, the tube voltage setting (80 kV) was applied for all the patients with an effective diameter lower than 22.7 cm. For patients with an effective diameter larger than 22.7 cm, a tube voltage of 80 kV (nine patients), 100 kV (eight patients), or 120 kV (one patient) was manually set by radiographers (Fig 1). In ATVS + ATCM group, the reference tube voltage setting (100kV) was not changed for patients with an effective diameter lower than 24.3 cm. For scans of patients with an effective diameter larger than 24.3 cm, either the reference tube voltage of 100 kV (four patients) or 120 kV (four patients) was set (Fig 1). In ATVS + the ATCM group, the tube voltage was not reduced to 80 kV in any of the scans.

If ATVS increased the tube voltage to 120 kV, the reference mAs was reduced from 80 to 50 by ATCM. Table 2 compares the mean value of kV, mAs, SDNR, and CTDI_vol_, including CTDI_vol_ ratio without and with ATVS, for five age groups. A slight increase in CTDI_vol_ was noticed in the age group 10-to-15 years (+15%); otherwise, CTDI_vol_ was reduced between 9 and 46%, approximately, depending on the age group (Table 2).

**Table 2:** Mean value of effective diameter (EDM), kV, mAs, SDNR, and CTDI_vol_ of chest CTs from the study age groups (32 cm PMMA phantom), with ATCM and with ATVS + ATCM.

Age group (years)	Patients’ number		EDM (cm)		Mean kV		Mean mAs		Mean SDNR		CTDI_vol_ (mGy)		CTDI_vol_ with ATVS and ATCM vs. CTDI_vol_ with ATCM
	ATCM	ATVS + ATCM	ATCM	ATVS + ATCM	ATCM	ATVS + ATCM	ATCM	ATVS + ATCM	ATCM	ATVS + ATCM	ATCM	ATVS + ATCM	
**Newborn**	0	0	-	-	-	-	-	-	-	-	-	-	-
**< 1 year**	6	6	13.40	11.81	80	100	72.67	17.83	11.05	8.64	1.16	0.63	- % 46
**1 to < 5 years**	22	16	15.93	15.54	80	100	80.05	32.13	11.37	10.74	1.27	1.11	- % 13
**5 to < 10 years**	16	12	18.74	18.89	80	100	86	36.83	10.99	10.62	1.37	1.25	- % 9
**10 to < 15 years**	31	19	21.56	22.29	81.94	101.05	95.19	56.11	10.91	10.29	1.71	1.96	+ %15
**≥ 15 (adult)**	17	15	23.57	23.26	88.24	104	101.35	49.33	10.03	9.04	2.42	1.91	- %21

EDM, Effective diameter; SDNR, Signal-difference-to-noise ratio; PMMA, polymethyl methacrylate; ATCM, automated tube current modulation; ATVS, automated tube voltage selection.

### CTDI_vol_ as a function of effective diameter

**Fig 2:**
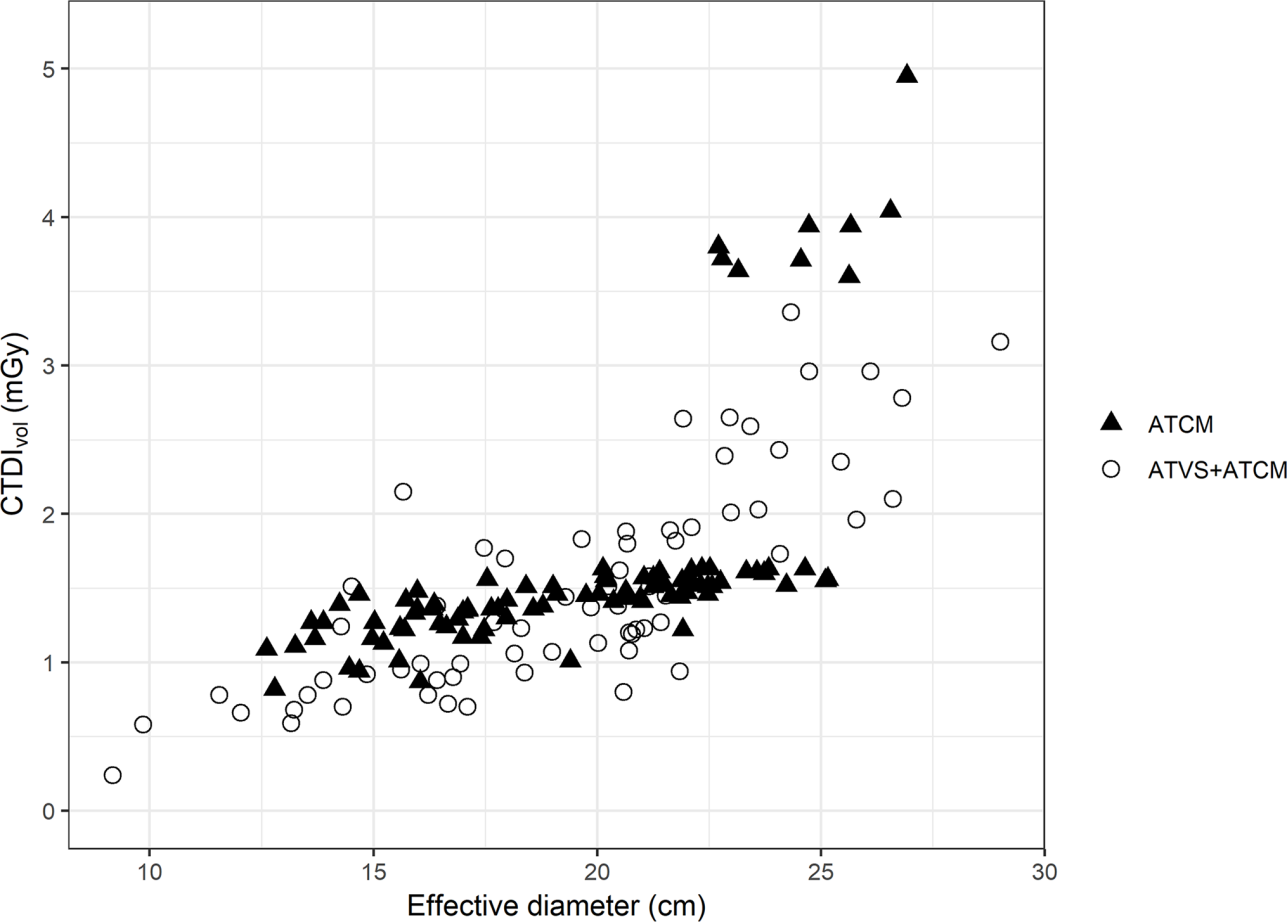
CTDI_vol_ (CTDI) as a function of effective diameter in the ATCM group and in the ATVS + ATCM group.

When looking at CTDI_vol_ as a function of effective diameter, a clear separation of the observations into two point-clouds was observed in the ATCM group, corresponding to the patient’s diameter (Fig 2). The point-cloud in the upper right corner corresponded to a manual increase in kV by the radiographers, without altering the effective (reference) mAs. With ATVS, fluctuations in CTDI_vol_ were reduced, and a better correlation of patients’ diameter with dose was seen, which can be recognized by the deeper and closer position of the circles in comparison to the triangles, also for younger and smaller patients’ group (Fig 2).

In the ATCM group, the correlation of CTDI_vol_ with effective diameter was r = 0.62 and, in the ATVS + ATCM group, r = 0.80 (difference without/with ATVS: -0.18; confidence interval -0.04 to -0.60; p = 0.025). This indicated a significantly larger correlation of CTDI_vol_ with effective diameter in the ATVS + ATCM group; however, the difference was rather small.

### Objective image quality without and with ATVS

**Fig 3:**
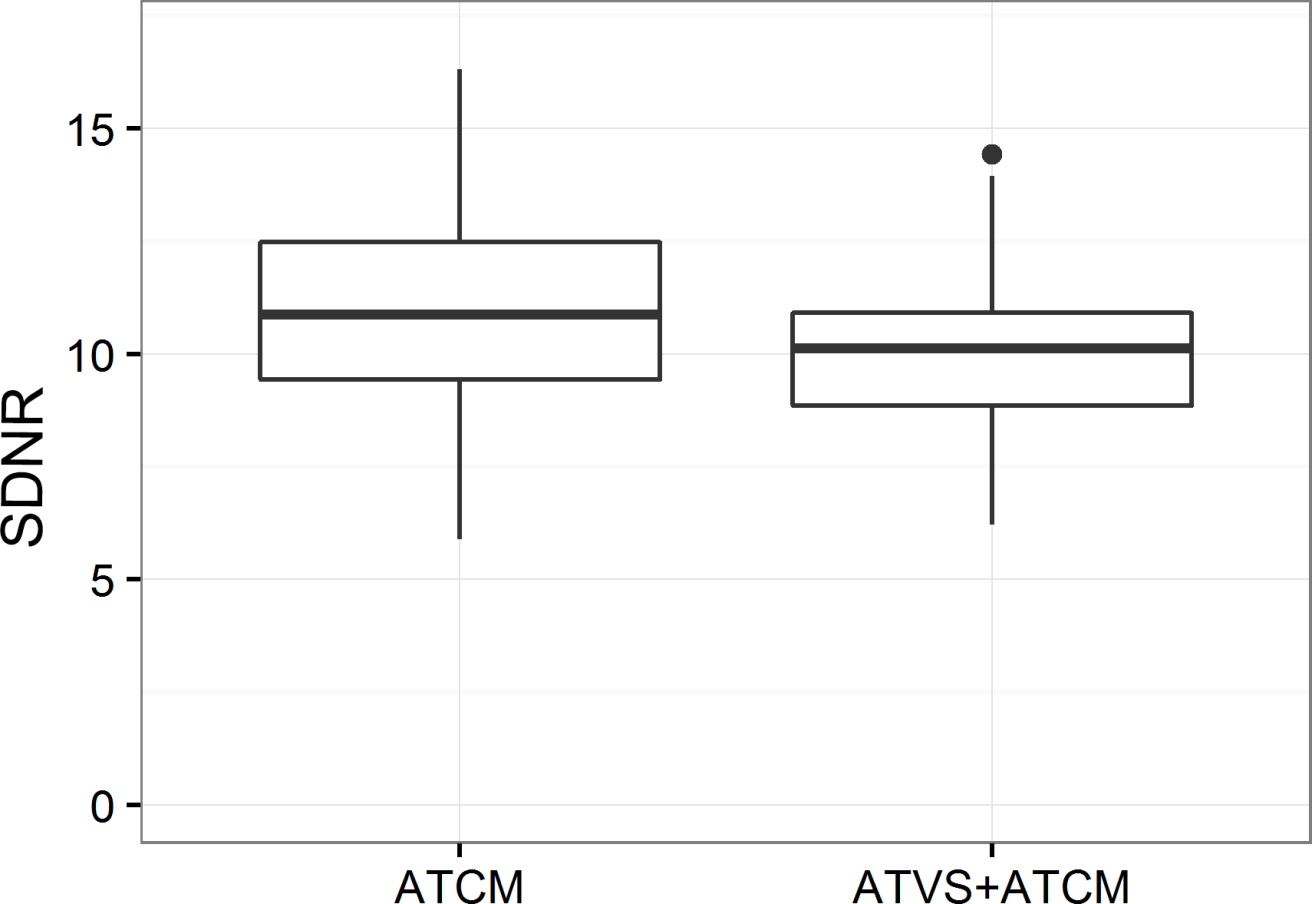
Boxplots of mean SDNR measurements in the ATCM group and in the ATVS + ATCM group.

Objective image quality was assessed by three SDNR measurements. The mean of these measurements was calculated. A two-sided, independent-samples t-test comparing mean SDNR without and with ATVS demonstrated a small, but significant decrease in mean SDNR with the use of ATVS (mean without ATVS: 10.88 vs mean with ATVS: 10.03, p = 0.0089, Fig 3).

### Agreement between the readers

**Fig 4:**
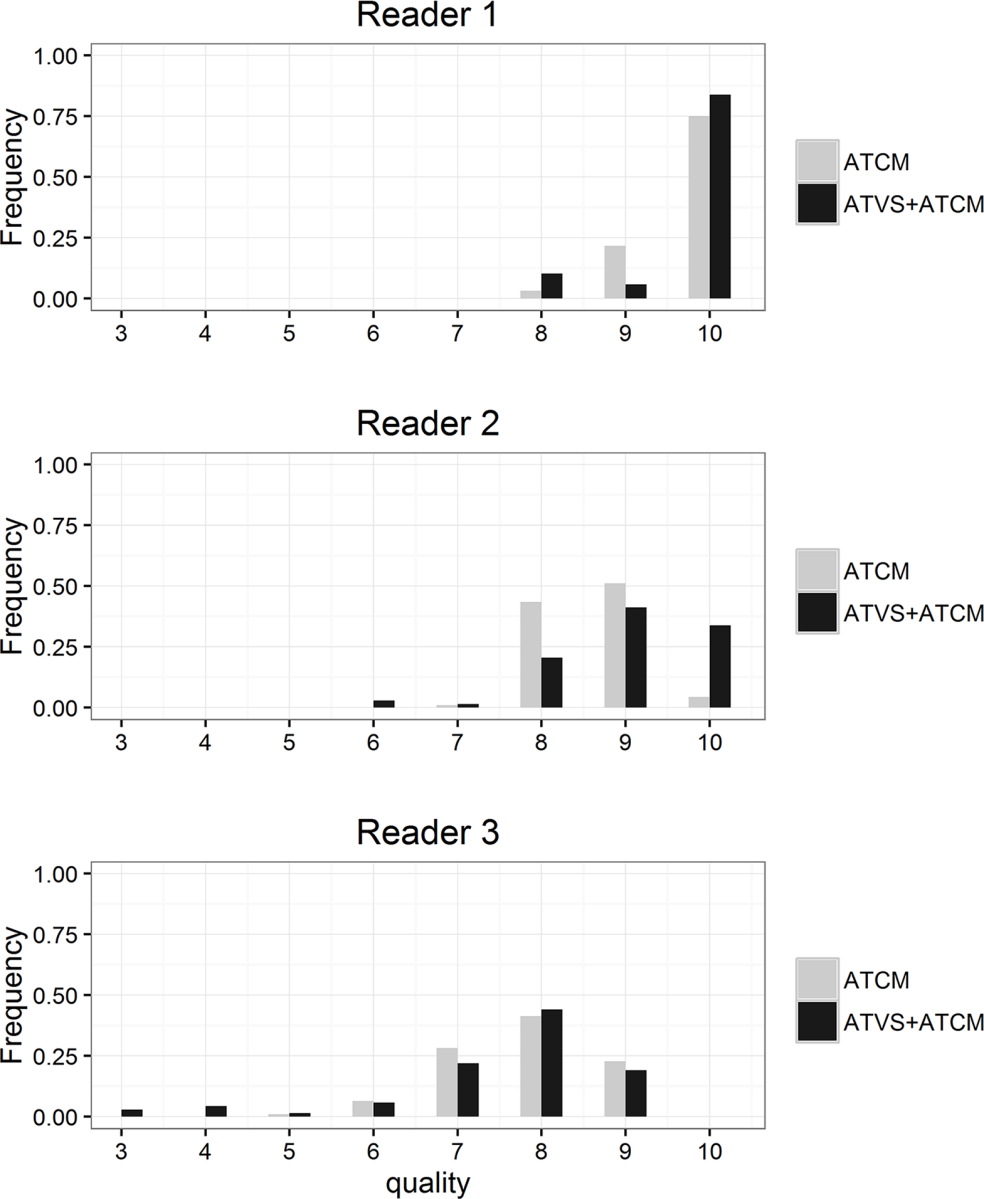
Distribution of image quality for each reader, both in the ATCM and in the ATVS + ATCM group.

The distribution of the ratings for each reader without and with ATVS is shown in Fig 4. Most ratings indicated higher quality (7–10 points), while very few images were rated below 7. Reader 3 (general radiologist) tended to provide lower ratings (often lower than 7), both without and with ATVS, compared to the two pediatric radiologists.

Table 3 shows weighted kappa coefficients for readers’ agreement, with regard to subjective quality. Although the readers’ agreement with and without ATVS was still slight to fair, an increase was observed with the use of ATVS (weighted kappa in the ATCM group: 0.1–0.22 and in the ATVS+ ATCM group: 0.12–0.36).

**Table 3:** Weighted kappa coefficients (95% CI) assessing agreement between the readers.

**Kappa**	**ATCM**	**ATVS + ATCM**
**Reader PR1 vs Reader PR2**	0.1 (0.03–0.16)	0.27 (0.09–0.45)
**Reader PR1 vs Reader GR**	0.1 (0.06–0.14)	0.12 (0.05–0.2)
**Reader PR2 vs Reader GR**	0.22 (0.11–0.34)	0.36 (0.24–0.49)

ATCM, automated tube current modulation; ATVS, automated tube voltage selection; PR1, pediatric radiologist with seven years experience; PR2, pediatric radiologist with eight years experience; GR, general radiologist with seven years experience.

**Fig 5:**
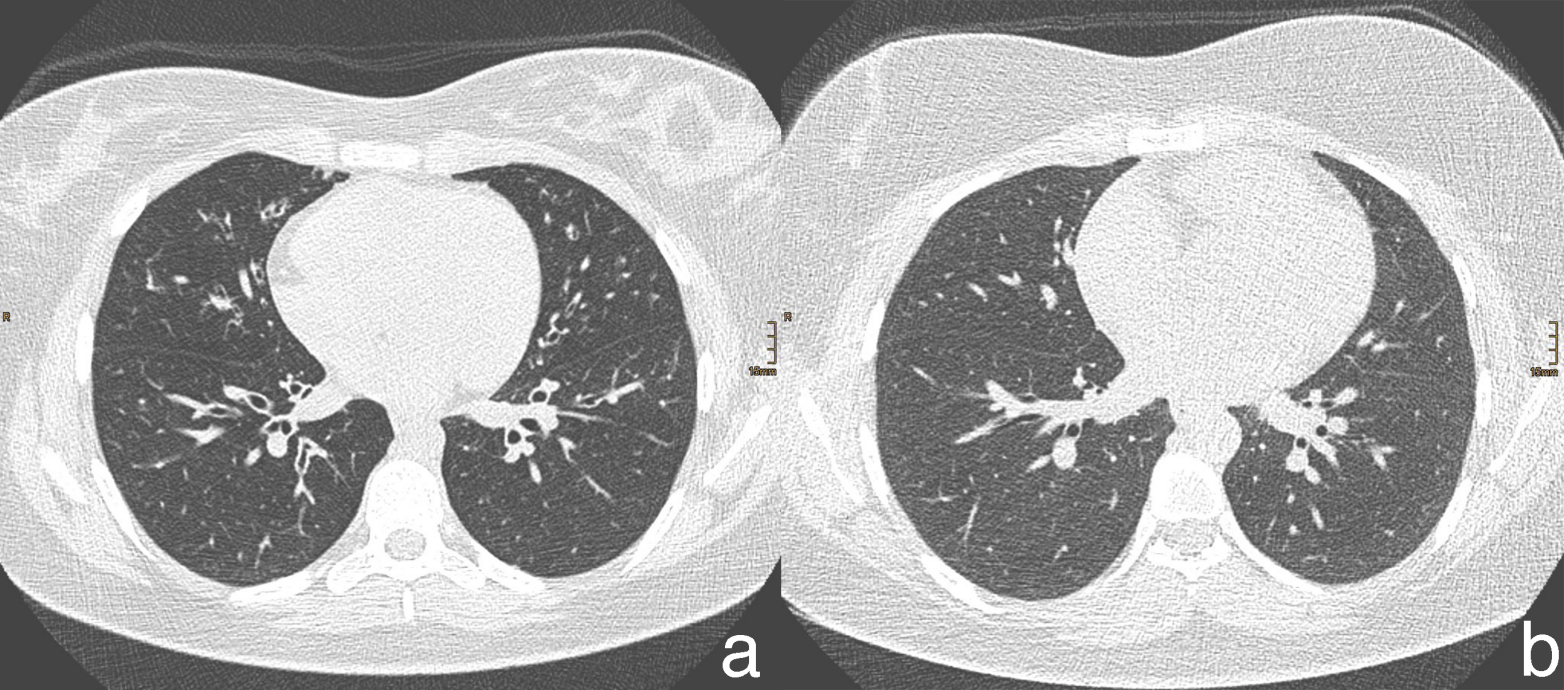
Two Examples of image quality for the chest scan a) with ATCM only and b) with ATVS + ATCM.

Fig 5 shows examples of image quality. Fig 5A shows a chest scan of a 15-year-old, 65 kg, 165 cm large girl. The effective diameter was 26.6 cm. Tube voltage was set to 100 kV, the reference tube current time product to 50 mAs, and only ATCM was activated. The effective tube current time product was 120 mAs, modulated by ATCM to a higher value of tube current time product, according to the patient’s size. The CTDI_vol_ was 4.04 mGy, and the dose length product (DLP) was 133 mGy.cm. The ratings of the two pediatric radiologists for the overall image quality were 10 and 9, respectively, and the rating of the general radiologist was 9. Fig 5B shows a chest scan, with activated ATCM and ATVS, of a 17-year-old, 64 kg, 151 cm large girl. The effective diameter was 29 cm. The reference tube voltage was set to 100 kV, and the reference tube current time product to 80 mAs. Based on the patient’s attenuation on the topogram, ATVS did not change the tube voltage. The effective tube current time product was 94 mAs, modulated by ATVS to a higher value of tube current time product, depending on the patient’s size. The CTDI_vol_ was 3.16 mGy, and the DLP was 104 mGy.cm. Both pediatric radiologists rated overall image quality as 10, and the rating of the general radiologist was 8. Both patients were of an almost equal weight, and the tube voltage of both scans was the same, but ATVS in combination with ATCM resulted in a smaller CTDI_vol_ (4.04 mGy vs. 3.16mGy).

## Discussion

We observed that ATVS, in combination with ATCM, was efficient in tailoring the tube voltage to the individual diameter of the patient. The overestimation of the necessary tube voltage, particularly in larger patients, led to violations of the in-house CT protocols by radiographers, and thus, to an increase in radiation dose (Fig 2). In the ATCM group, there were two acquisition protocols with two different tube voltages (80 kV and 100 kV), depending on the patient weight, and radiographers in our hospital tended to manually increase kV to adjust for higher patient weights without adjusting reference mAs for the tube current modulation accordingly, which resulted in unnecessarily high radiation doses for some larger children. These suboptimal adjustments appeared as a distinct point-cloud in the top right corner in Fig 2. But, in the ATVS + ATCM group, a smoother and more reasonable relation between CTDI_vol_ and effective diameter was observed, and CTDI_vol_ outliers, which were shown in larger and/or older patients, were reduced (Fig 2). Previous publications have also shown that ATVS is most useful and efficient in combination with ATCM [18, 19]. Thus, the main effect of ATVS, in combination with ATCM, on radiation dose is mainly a homogenization of CT protocols. Although one may speculate that the same effect may be achieved by continuous personnel training, ATVS, in combination with ATCM, appears to be the more efficient tool to achieve the goal of keeping the radiation dose as low as is reasonably achievable.

We could demonstrate that ATVS, in combination with ATCM, did reduce the radiation dose in non-contrast pediatric chest CT in four of five age groups. In particular, for the smallest patients (≤1 year), CTDI_vol_ was reduced by approximately 50 percent, and CTDI_vol_ for the largest group (≥15 years) by approximately 20 percent. In patients between one and 10 years of age, ATVS resulted in just a slight decrease in CTDI_vol_ by approximately 10 percent. In the 10-to-15-year-old age group, there was a slight increase in CTDI_vol_ (+15%), which was paralleled by a small increase of the mean value of patients’ effective diameter of 3.39% (Table 2). But, in the age groups less than one 1 year, 1 to < 5 years, and ≥15 years, the mean values of the patients’ effective diameter decreased by 11.87%, 2.45%, and 1.32%, respectively. In the age group 5 to <10 years, there was a very slight increase in the mean value of the patients’ effective diameter of 0.8% (Table 2). This could also be seen in the significantly better correlation of CTDI_vol_ with effective diameter in the ATVS + ATCM group; the correlation of CTDI_vol_ with effective diameter was r = 0.62 in the ATCM group and, in the ATVS + ATCM group, r = 0.80; p = 0.025.

These results are in contrast to a study by Spearman et al. [29], who evaluated the effect of ATVS on CT radiation dose, including all body regions and types of CT examinations in 164,323 unique CTs from all across the world. They observed, in non-enhanced chest CTs, a non-significant decrease in CTDI_vol_ of -2.2%, but a significant decrease for enhanced chest CT of -14.3% [29]. In their study, however, they were not able to verify whether ATVS was consistently applied correctly with the appropriate settings [29].

We pooled patient dose data in age groups, in accordance with the recommended procedures of the IAEA and the ASI, for comparing children’s doses to dose reference levels using age-banding [25, 26], and because the lifetime risk of cancer incidence and cancer mortality depends on the age at exposure [6]. The longer life expectancy of children and the higher radiation sensitivity during organ development, during which time a cancer can become established and develop, reflect the increased risk for a given dose for younger age groups [25].

ATVS uses attenuation information collected by the topogram, and selects kV and modulates mAs to maintain the user-chosen image quality [12, 17, 20]. Therefore, we correlated CTDI_vol_ with effective diameter in our study to evaluate the effect of ATVS, and measured at the widest area in the axial CT thoracic scans, which almost always corresponded to the thoracic diameter at the level of the 9th thoracic vertebral body.

In our study, automatic tube current modulation was applied in both groups, without and with ATVS. We observed that ATVS, in combination with ATCM did not reduce the reference tube voltage of 100 kV to 80 kV for patients with an effective diameter lower than 24.3 cm, as in the manual tube voltage selection in the ATCM group. For patients with an effective diameter higher than 24.3, ATVS selected 120 kV. If ATVS increased the tube voltage to 120 kV, the reference mAs was reduced from 80 to 50 by ATCM. Increasing the kV improves the soft-tissue contrast noise levels of a patient’s anatomy on non-contrast CT scans, which raises the patient’s radiation dose, but, with appropriate mAs reduction, this results in reasonable patient doses [10]. The choice of kV should be made on the basis of the need for sufficient contrast in the image, as well as on subject size [30]. This is how ATVS, in combination with ATCM, works. Dose reduction in non-contrast CT scans is achieved by setting the DSO to 3. At DSO 3, ATVS increases the tube voltage for larger patients—depending on the attenuation of the topogram—for a better soft-tissue contrast noise level, thereby increasing the patient's dose. But, in combination with ATCM, a patient’s radiation dose is reduced by a reduction of the reference tube current time product. We observed this effect in our study collective—while ATVS increased the tube voltage from 100 kV to 120 kV, the reference mAs setting was reduced from 80 to 50 by ATCM.

However, CTDI_vol_ for patients younger than 10 years was decreased (9–46%) in the ATVS + ATCM group, by lowering mAs below the reference setting, compared to the ATCM group, in which the radiographer selected the tube voltage manually, without adjusting the reference mAs. This could be explained by setting the DSO on level 3, as suggested for non-contrast examinations. The ATVS system investigated in this study (CarekV, Siemens Healthineers, Siemens Healthcare GmbH, Germany) provides 12 modulation levels (dose saving optimized, DSO, levels), which can be selected by the user, depending on the diagnostic task [17]. For example, DSO 1 (equal noise) aims to achieve an equal noise level at a lower dose compared to the reference tube voltage; in unenhanced lung CT, this level mostly results in the selection of a higher tube voltage than the reference tube voltage, with very low mAs [17]. At the other end of the scale, DSO 12 aims to achieve an equal contrast-to-noise-ratio (CNR), compared to the CNR of the reference tube voltage, by lowering the tube voltage with low mAs settings [17]. CT angiography applications are usually performed using a DSO 12, as, in such examinations, a higher image noise can be tolerated and will benefit from the high iodine signal at the lower kV [17]. In non-contrast exams, there is no quality benefit from lowering kV, leading to rising noise and image quality loss [20]. At DSO level 3, ATVS, in combination with ATCM, selects kV and modulates mAs to optimize image quality, depending on the patient attenuation, measured by the topogram [20]. As a consequence, ATVS does not alter the reference kV for smaller children and dose reduction is achieved by ATCM alone. In larger children, ATVS increases kV and ATCM modulates the mAs in relation to the reference settings.

In our study, ATVS, in combination with ATCM, resulted in a small, but significant decrease in mean SDNR compared to the ATCM group (p = 0.0089). The number of patients in our study was too small to demonstrate any correlation between SDNR and the subjective image quality in each age group. We could demonstrate, however, that the reader agreement increased with ATVS implementation, even though the SDNR decreased after ATVS implementation (Tables 2 and 3 and Figs 3 and 4). However, the readers in our study did not perceive a loss of image quality (Fig 4). Siegel et al. [19] observed that the use of ATVS in contrast-enhanced pediatric CT and CT angiography lowered the radiation dose and while maintaining image quality. Frellesen et al. [17] showed a consistent image quality for thoraco-abdominal trauma CT after the implementation of ATVS. Although the general radiologist in our study rated pediatric chest CT scans lower than the two pediatric radiologists, she did not report any significant image quality change in scans with ATVS (Fig 4). The agreement between the readers increased with ATVS, albeit slightly (Table 3). The lower image quality ratings might thus reflect the lesser experience of the general radiologist with pediatric chest CTs, compared to dedicated pediatric radiologists.

### Limitations

Although our retrospective study was based on a limited number of cases (160 chest scans), the results are still convincing. Other publications, which evaluated image quality and radiation exposure after ATVS implementation, also had to rely on small sample sizes [12, 14, 19]. All of those studies observed a radiation reduction, while maintaining [12, 19] or improving [14] image quality.

The inherent interobserver variability between the general radiologist and the two pediatric radiologists could be an institution-specific situation and could be improved if more training is offered for readers of pediatric CT imaging.

## Conclusion

An appropriate automated tube voltage selection, combined with automatic tube current modulation, prevents errors in manual tube voltage setting and protects older and/or larger children against an unnecessarily high radiation exposure and results in a radiation dose reduction for pediatric non-contrast chest CT up to a factor of about 2, without a loss of diagnostic image quality.

## Supporting information

S1 Data (PDF)
