# Supplementary material for: Automated tube voltage selection in pediatric non-contrast chest CT
Source: PLoS One. 2018 Oct 3;13(10):e0204794. doi: 10.1371/journal.pone.0204794 (PMC6169939; doi:10.1371/journal.pone.0204794)
Supplement: S1 Data — (PDF) [file pone.0204794.s001.pdf]

				Exposure Parameters				Objective Image Quality	Subjective Image Quality				
No	Sex	Age years	Effective Diameter cm	kV	mAs ref	mAseff	CTDI	Mean SNR	PR1	PR2	GR	Comment	
1	male	6	17,1	80	50	85	1,35	12,82608317	10	9	9		ATCM
2	female	15	26,55	100	50	120	4,04	8,014579173	10	9	9		ATCM
3	male	3	17	80	50	74	1,17	10,67632592	10	9	8		ATCM
4	male	15	22,56	80	50	95	1,51	9,107199276	10	9	9		ATCM
5	male	4	18,41	80	50	94	1,51	9,508892496	9	9	7		ATCM
6	male	0	14,24	80	50	88	1,39	11,31736876	9	9	7		ATCM
7	male	10	21,25	80	50	95	1,51	11,66630048	10	10	8		ATCM
8	female	3	14,46	80	50	60	0,96	7,092733254	9	9	6		ATCM
9	male	15	25,15	80	50	98	1,56	8,204147181	10	10	9		ATCM
10	female	8	17,39	80	50	73	1,17	7,748644643	10	10	7		ATCM
11	male	12	21,39	80	50	102	1,61	6,697654295	10	9	7		ATCM
12	male	6	19,4	80	50	64	1,01	7,55354021	10	8	8		ATCM
13	male	15	22,47	80	50	92	1,46	8,805154245	10	8	8		ATCM
14	male	11	24,73	100	50	118	3,94	14,13253764	10	9	9		ATCM
15	female	6	17,54	80	50	98	1,56	10,69016604	10	8	8		ATCM
16	female	3	15,98	80	50	86	1,36	14,26379202	9	8	6		ATCM
17	male	2	16,48	80	50	79	1,26	11,53260034	9	8	7		ATCM
18	male	11	21,25	80	50	99	1,57	10,34975802	9	9	8		ATCM
19	female	10	18,57	80	50	86	1,36	13,58991272	10	8	8		ATCM
20	female	16	22,52	80	50	102	1,63	7,169532855	8	9	8		ATCM
21	male	9	23,73	80	50	100	1,6	8,564435076	10	9	9		ATCM
22	female	3	15,57	80	50	64	1,01	11,24383506	10	8	7		ATCM
23	male	16	24,55	100	50	112	3,71	14,13899863	10	9	9		ATCM
24	female	8	20,04	80	50	92	1,46	13,79655616	10	9	9		ATCM
25	female	0	12,79	80	50	50	0,82	10,428854	9	8	6		ATCM
26	male	12	22,02	80	50	96	1,53	9,080742337	10	9	8		ATCM
27	male	0	13,61	80	50	80	1,27	12,93858055	10	8	7		ATCM
28	male	8	21,89	80	50	98	1,55	9,758703498	9	8	8		ATCM
29	male	2	15,02	80	50	80	1,27	12,91081877	10	9	6		ATCM
30	male	7	17,1	80	50	86	1,36	12,99306233	9	8	7		ATCM
31	male	1	13,69	80	50	73	1,16	12,23907161	10	8	7		ATCM

32	male	14	22,79	100	50	112	3,72	16,31065864	10	9	9		ATCM
33	male	8	17,97	80	50	82	1,3	9,634964178	10	8	7		ATCM
34	male	15	21,47	80	50	96	1,52	10,25295728	10	8	9		ATCM
35	male	15	25,63	100	50	108	3,6	14,84735178	10	9	9		ATCM
36	female	14	25,66	100	50	118	3,94	11,0296365	10	9	8		ATCM
37	male	9	20,96	80	50	90	1,43	12,19387381	10	9	8		ATCM
38	male	5	16,88	80	50	80	1,29	11,44606623	10	8	7		ATCM
39	female	15	21,04	80	50	98	1,57	9,423959568	10	8	8		ATCM
40	female	4	17	80	50	84	1,34	13,78555776	10	8	7		ATCM
41	male	0	13,25	80	50	70	1,11	12,09151757	9	8	7		ATCM
42	male	4	15,69	80	50	76	1,22	11,07270063	10	8	7		ATCM
43	male	13	22,47	80	50	95	1,51	11,3340225	10	9	9		ATCM
44	female	2	17,78	80	50	86	1,36	9,614669245	10	8	8		ATCM
45	female	1	15,72	80	50	90	1,42	12,77873874	10	8	8		ATCM
46	female	10	20,36	80	50	89	1,41	10,3762342	10	9	7		ATCM
47	female	11	23,33	80	50	102	1,61	8,889772257	10	9	9		ATCM
48	female	12	22,31	80	50	96	1,52	10,97006101	10	9	9		ATCM
49	female	12	19,75	80	50	92	1,45	13,70447488	10	9	9		ATCM
50	female	10	18,78	80	50	86	1,38	11,78145564	10	9	9		ATCM
51	male	2	14,97	80	50	73	1,16	12,413097	9	8	7		ATCM
52	male	16	23,15	100	50	109	3,64	15,90938756	10	9	9		ATCM
53	female	16	22,34	80	50	102	1,63	6,484290865	9	9	6		ATCM
54	male	0	12,61	80	50	68	1,09	10,20043563	9	8	5		ATCM
55	male	2	14,68	80	50	92	1,46	5,895042314	9	8	7		ATCM
56	male	2	16,36	80	50	88	1,39	11,90435525	10	8	9		ATCM
57	male	2	15,22	80	50	72	1,13	10,53489346	9	8	8		ATCM
58	male	0	13,88	80	50	80	1,27	9,340144416	9	8	7		ATCM
59	female	11	16,04	80	50	54	0,87	10,48589352	10	9	8		ATCM
60	female	2	15,98	80	50	94	1,48	12,60809673	8	8	7		ATCM
61	female	14	23,57	80	50	102	1,61	8,122404372	10	9	8		ATCM
62	male	2	16,32	80	50	86	1,36	13,37558951	10	8	8		ATCM
63	female	11	21,65	80	50	92	1,45	12,72287092	10	9	8		ATCM
64	male	11	21,85	80	50	90	1,44	12,12851954	10	9	8		ATCM
65	male	2	14,68	80	50	59	0,94	11,63597321	9	7	7		ATCM
66	male	5	19,1	80	50	92	1,46	11,97092424	10	9	8		ATCM
67	female	15	24,64	80	50	102	1,63	8,680234488	10	9	8		ATCM

68	female	13	22,1	80	50	102	1,62	9,515338149	10	9	8		ATCM
69	female	2	15,92	80	50	84	1,33	9,308497894	10	8	7		ATCM
70	female	17	23,83	80	50	102	1,63	7,174818415	9	8	7		ATCM
71	male	16	26,92	120	110	73	4,95	10,16317088	10	10	9		ATCM
72	male	2	15,59	80	50	77	1,23	13,4017876	10	8	8		ATCM
73	female	14	21,91	80	50	76	1,22	13,30188269	10	9	8		ATCM
74	male	13	22	80	50	92	1,47	9,566998065	10	9	9		ATCM
75	male	10	20,19	80	50	98	1,55	8,864950687	10	8	7		ATCM
76	male	10	20,65	80	50	90	1,43	13,20243222	10	9	8		ATCM
77	female	15	20,13	80	50	102	1,63	8,776705555	9	9	8		ATCM
78	male	14	24,23	80	50	96	1,52	10,46626068	10	9	8		ATCM
79	female	15	22,71	100	50	114	3,8	13,9572966	10	9	8		ATCM
80	female	12	21,37	80	50	98	1,55	10,28464189	10	9	7		ATCM
81	male	15	25,1	80	50	98	1,55	9,437702604	10	8	8		ATCM
82	female	11	22,1	80	50	98	1,55	10,34269479	9	8	8		ATCM
83	female	12	21,01	80	50	89	1,41	10,96982951	10	8	8		ATCM
84	female	13	21,41	80	50	98	1,57	10,81039475	10	9	7		ATCM
85	female	13	22,75	80	50	97	1,54	10,05238474	10	9	8		ATCM
86	female	7	17,47	80	50	76	1,22	12,67159066	10	8	8		ATCM
87	female	7	16,63	80	50	78	1,24	11,15197719	10	8	8		ATCM
88	female	12	20,64	80	50	93	1,48	10,95499007	10	9	8		ATCM
89	male	7	17,63	80	50	86	1,36	12,37202158	10	9	9		ATCM
90	male	9	19,01	80	50	96	1,51	10,41253205	10	8	8		ATCM
91	female	13	20,18	80	50	100	1,58	6,631141667	8	8	6		ATCM
92	male	4	17,98	80	50	90	1,42	12,43208222	10	9	7		ATCM
93	male	2	16,67	100	80	20	0,72	10,27134607	10	9	5	ATVS	ATCM
94	male	7	19,65	100	80	54	1,83	12,10278546	10	10	7	ATVS	ATCM
95	male	9	20,67	100	80	54	1,8	8,150730737	10	9	7	ATVS	ATCM
96	female	14	20,02	100	80	34	1,13	7,69161614	10	9	8	ATVS	ATCM
97	female	15	21,85	100	80	28	0,94	6,344072109	10	9	8	ATVS	ATCM
98	male	14	25,8	100	80	58	1,96	12,46096868	10	10	8	ATVS	ATCM
99	male	4	16,42	100	80	40	1,38	14,43207042	10	9	7	ATVS	ATCM
100	female	1	13,53	100	80	22	0,78	12,00777429	8	8	4	ATVS	ATCM
101	male	8	18,15	100	80	32	1,06	12,80673032	9	9	8	ATVS	ATCM
102	male	1	14,51	100	80	44	1,51	13,95348037	10	8	7	ATVS	ATCM
103	female	15	24,07	100	80	72	2,43	9,257466417	10	9	8	ATVS	ATCM

104	male	3	17,47	100	80	52	1,77	13,80492928	10	9	7	ATVS	ATCM
105	male	11	20,78	100	80	34	1,19	10,34048739	10	9	8	ATVS	ATCM
106	male	5	16,78	100	80	26	0,9	9,9396257	10	9	8	ATVS	ATCM
107	male	4	17,69	100	80	38	1,27	9,151138919	10	10	8	ATVS	ATCM
108	female	15	20,59	100	80	24	0,8	7,430666449	10	9	7	ATVS	ATCM
109	male	10	25,45	100	80	70	2,35	10,67198688	10	10	9	ATVS	ATCM
110	female	2	15,67	100	80	64	2,15	10,26249176	10	8	7	ATVS	ATCM
111	female	4	16,42	100	80	26	0,88	11,20269561	10	9	8	ATVS	ATCM
112	male	13	22,11	100	80	56	1,91	12,69918083	10	10	8	ATVS	ATCM
113	male	6	18,99	100	80	32	1,07	10,32601774	9	9	7	ATVS	ATCM
114	male	13	21,92	100	80	80	2,64	9,299928825	10	9	8	ATVS	ATCM
115	male	17	26,11	120	50	50	2,96	10,54032087	10	10	8	ATVS	ATCM
116	male	11	20,47	100	80	40	1,38	10,32477064	10	10	8	ATVS	ATCM
117	male	8	20,71	100	80	32	1,08	8,334404126	10	10	9	ATVS	ATCM
118	male	11	21,63	100	80	56	1,89	7,196776282	10	9	8	ATVS	ATCM
119	female	4	16,94	100	80	30	0,99	9,991273747	10	10	7	ATVS	ATCM
120	female	12	19,86	100	80	40	1,37	12,73148184	10	10	8	ATVS	ATCM
121	male	3	16,22	100	80	22	0,78	9,987726234	8	8	4	ATVS	ATCM
122	female	0	13,16	100	80	17	0,59	8,738908845	10	8	6	ATVS	ATCM
123	female	15	21,75	100	80	54	1,82	10,90175734	10	9	9	ATVS	ATCM
124	female	0	13,23	100	80	20	0,68	10,70348327	10	8	6	ATVS	ATCM
125	male	13	23,42	100	80	80	2,59	9,458703461	9	9	6	ATVS	ATCM
126	female	0	11,55	100	80	22	0,78	8,347575791	9	7	3	ATVS	ATCM
127	male	0	13,88	100	80	26	0,88	10,81601899	10	8	7	ATVS	ATCM
128	female	15	21,53	100	80	42	1,45	9,786982985	10	10	9	ATVS	ATCM
129	female	13	24,33	120	50	60	3,36	8,881270356	8	9	8	ATVS	ATCM
130	male	13	22,96	100	80	80	2,65	10,46409472	8	9	7	ATVS	ATCM
131	male	4	15,62	100	80	28	0,95	10,72736578	10	8	8	ATVS	ATCM
132	female	13	20,87	100	80	36	1,22	9,140511064	10	9	9	ATVS	ATCM
133	male	11	22,99	100	80	60	2,01	11,10321966	10	10	9	ATVS	ATCM
134	female	11	22,85	100	80	72	2,39	10,96773411	10	10	8	ATVS	ATCM
135	male	3	14,28	100	80	36	1,24	10,63530151	8	8	6	ATVS	ATCM
136	male	17	26,82	120	50	50	2,78	10,02712561	10	10	8	ATVS	ATCM
137	male	6	18,38	100	80	28	0,93	11,77264693	10	9	8	ATVS	ATCM
138	male	14	26,61	100	80	62	2,1	12,08437919	10	10	9	ATVS	ATCM
139	female	5	17,1	100	80	20	0,7	9,820333685	10	10	8	ATVS	ATCM

140	male	4	16,05	100	80	28	0,99	8,396553209	8	8	7	ATVS	ATCM
141	male	11	20,25	100	80	44	1,51	6,205400709	10	9	8	ATVS	ATCM
142	female	16	21,42	100	80	38	1,27	6,90990354	10	8	9	ATVS	ATCM
143	female	2	14,85	100	80	26	0,92	8,369448995	10	8	7	ATVS	ATCM
144	male	9	18,3	100	80	36	1,23	11,17723893	10	9	8	ATVS	ATCM
145	male	9	17,94	100	80	50	1,7	10,48298758	10	10	8	ATVS	ATCM
146	female	2	14,31	100	80	20	0,7	9,243522797	10	8	7	ATVS	ATCM
147	male	17	24,09	100	80	52	1,73	9,855973719	10	10	8	ATVS	ATCM
148	male	16	21,17	100	80	44	1,51	10,23229594	10	9	8	ATVS	ATCM
149	male	10	20,65	100	80	56	1,88	10,28489697	10	10	9	ATVS	ATCM
150	male	16	21,17	100	80	46	1,58	9,34075777	10	9	8	ATVS	ATCM
151	female	16	24,74	120	50	50	2,96	8,664725269	10	10	9	ATVS	ATCM
152	female	1	12,04	100	80	18	0,66	9,46475564	10	8	7	ATVS	ATCM
153	female	0	9,85	100	100	16	0,58	6,537572319	8	6	4	ATVS	ATCM
154	female	16	23,6	100	80	60	2,03	8,705648492	10	9	9	ATVS	ATCM
155	male	12	20,5	100	80	48	1,62	13,53178751	10	9	8	ATVS	ATCM
156	female	0	9,17	100	100	6	0,24	6,713741934	10	6	3	ATVS	ATCM
157	male	9	20,71	100	80	36	1,2	10,65114964	10	9	9	ATVS	ATCM
158	female	17	29,01	100	80	94	3,16	8,09995523	10	10	8	ATVS	ATCM
159	male	6	19,29	100	80	42	1,44	11,85096752	10	10	8	ATVS	ATCM
160	female	15	21,05	100	80	36	1,23	9,494935038	10	10	9	ATVS	ATCM
